# Enhanced Corrosion Resistance of Waterborne Epoxy Coatings by High-Entropy Layered Double Hydroxides/Graphitic Carbon Nitride Fillers

**DOI:** 10.3390/ma19122576

**Published:** 2026-06-15

**Authors:** Shaolei Song, Xin Chen, Peiqi Jiang, Wenchang Liang, Yuanyuan Liu, Dongjiang Pan, Qing Guo, Lei Lei, Yan Li

**Affiliations:** 1 School of Civil Engineering, Xuzhou University of Technology, Xuzhou 221018, China; 2 Analysis and Testing Center, Xuzhou University of Technology, Xuzhou 221018, China; 3 School of Materials and Chemical Engineering, Xuzhou University of Technology, Xuzhou 221018, China

**Keywords:** Q235 steel, waterborne epoxy coating, HE-LDHs/g-C_3_N_4_ filler, corrosion resistance

## Abstract

Two-dimensional nanomaterials exhibit excellent physical barrier properties, which can effectively enhance the corrosion resistance of waterborne epoxy coatings. Herein, we report a facile strategy for preparing a multi-component synergistic anti-corrosion coating, where two-dimensional graphitic carbon nitride (g-C_3_N_4_) and high-entropy layered double hydroxides (HE-LDHs) are integrated into a waterborne epoxy matrix via magnetic-ultrasonic synergistic dispersion. The resulting HE-LDHs/g-C_3_N_4_-epoxy coating exhibits exceptional corrosion resistance for Q235 steel. Electrochemical impedance spectroscopy (EIS) and polarization curves showed that when the mass ratio of g-C_3_N_4_ to HE-LDHs was 1:1, the resulting coating (PCN-LDH-1.0) maintained a coating resistance of 5.48 × 10^5^ Ω·m^2^ after 28 days of immersion in 3.5% NaCl solution, which was five orders of magnitude higher than that of pure waterborne epoxy coating. Meanwhile, the corrosion current density was reduced by four orders of magnitude, from 5.83 × 10^−1^ A·m^−2^ to 1.68 × 10^−5^ A·m^−2^. After 30 days of salt spray testing, no rust, blistering or adhesion loss was observed on the coating surface. These enhanced performances by addition of g-C_3_N_4_ and HE-LDHs were attributed to the combined effects of the tortuous diffusion pathways. Additionally, the PCN-LDH-1.0 coating retained excellent mechanical properties, including a pencil hardness of 3H and the highest adhesion grade. This study provides a facile method for preparing high-performance waterborne anti-corrosion coatings.

## 1. Introduction

With the acceleration of industrialization, metallic materials have been increasingly deployed in diverse fields such as marine engineering, petrochemical industry, and infrastructure construction [1]. However, corrosion of metallic materials has become a pivotal constraint restricting their service life and safety performance [2]. Unlike solvent-based counterparts that pose environmental and health risks, waterborne epoxy coatings have been widely used for corrosion protection of metals due to their excellent barrier properties, superior adhesion strength compared to traditional coatings, and low volatile organic compound (VOC) emissions [3,4]. Nevertheless, micro-pores are inevitably formed in the coating during solvent evaporation and epoxy prepolymer curing/crosslinking processes, which provide channels for the intrusion of corrosive media, leading to a decline in coating barrier performance and a significant reduction in service life [5]. Therefore, there is an urgent need to develop a strategy that can enhance the corrosion resistance of epoxy coatings against corrosive media without compromising their inherent advantages.

To enhance the corrosion resistance of waterborne epoxy coatings, researchers have proposed a plethora of modification strategies [6]. Among them, nanofiller modification has been recognized as an effective and straightforward approach, capable of improving the barrier properties of coatings while simultaneously enhancing other performance attributes [7,8,9]. Among a wide range of nanofillers, two-dimensional (2D) nanofillers have exhibited outstanding barrier performance in corrosion protection coatings in comparison with one-dimensional or zero-dimensional nanofillers, attributed to their high specific surface area, lamellar structure, and excellent chemical corrosion resistance, which can effectively block and resist the penetration of corrosive media [10,11,12,13]. Particularly in waterborne epoxy systems, 2D nanofillers can better integrate with the aqueous matrix due to their lamellar structure, further enhancing the modification effect. Graphitic carbon nitride (g-C_3_N_4_), as a graphene-like two-dimensional layered material, has become a promising candidate filler for coatings [4]. It exhibits a molecular-scale barrier effect analogous to graphene and exceptional barrier properties. This is primarily attributed to its unique electronic structure and high electronegativity. The layered structure of g-C_3_N_4_, combined with its abundant nitrogen atoms, creates a barrier that impedes the movement of electrons [4]. This barrier effect reduces the electrochemical activity on the metal surface, thereby decreasing the rate of electrochemical corrosion [3,11,14]. Li et al. [14] incorporated highly crystalline carbon nitride synthesized via thermal polymerization into epoxy resin. The resulting coating exhibited a remarkable barrier effect, demonstrating excellent corrosion resistance efficiency and long-term service life. Guo et al. [15] synthesized N-defective g-C_3_N_4_ through a simple base-assisted thermal polymerization method, and the material showed excellent corrosion resistance. He et al. [16] combined platelet g-C_3_N_4_ with graphene nanoplatelets and incorporated them into a waterborne epoxy system. The two-dimensional lamellar structure of these fillers formed a “tortuous path effect” within the coating, significantly prolonging the diffusion path of corrosive media and effectively enhancing the barrier properties of the coating. EIS tests revealed that after 60 days of immersion, the impedance modulus of the g-C_3_N_4_@GO/epoxy coating remained at 5.23 × 10^3^ Ω·m^2^, which was substantially higher than that of the pristine epoxy coating (1.60 × 10^0^ Ω·m^2^), with a difference of three orders of magnitude. These studies collectively demonstrate the great potential of g-C_3_N_4_ in enhancing coating corrosion resistance, yet they all overlook the critical issue of dispersion uniformity in waterborne systems. Regrettably, due to the extremely high specific surface area and strong interlayer adhesion forces of g-C_3_N_4_ nanosheets, the uniformity of its aqueous dispersion falls far short of expectations [17]. It tends to aggregate in waterborne epoxy matrices, failing to fully exert its barrier and protective functions, which significantly diminishes the corrosion resistance of the coating [18,19].

Layered double hydroxides (LDHs) are a category of inorganic nanomaterials with a lamellar structure, where the interlayer spaces can accommodate various anions or water molecules [20,21]. Owing to their unique lamellar architecture and tunable interlayer chemical properties, LDHs have demonstrated application prospects in fields such as catalysis, adsorption, and barrier protection [22,23,24,25,26]. Building on the lamellar structure advantage of LDHs, the emerging high-entropy concept has been introduced to develop a new generation of functional nanomaterials. High-entropy materials, as a burgeoning research frontier, have shown great application potential in protective coatings and other fields, attributed to their unique advantages such as the high-entropy effect and cocktail effect [27]. High-entropy layered double hydroxides (HE-LDHs) are ingeniously engineered by incorporating multiple metal cations into the lamellar structure of conventional LDHs, uniquely endowing the material with both the structural merits of layered materials and the core characteristics of high-entropy systems [28]. The surface hydroxyl groups of HE-LDHs significantly augment hydrophilicity and interfacial activity, enabling uniform dispersion in aqueous systems, while also forming robust interfacial bonds with the resin matrix through hydrogen bonding and electrostatic interactions, thereby dramatically enhancing the coating’s corrosion resistance and mechanical properties [29]. The synergistic combination of HE-LDHs with g-C_3_N_4_, where the lamellar HE-LDH structure acts as both a physical barrier and a dispersion template, effectively isolates g-C_3_N_4_ nanosheets to prevent aggregation within the resin matrix, while coordinative interactions between HE-LDHs hydroxyls and g-C_3_N_4_ nitrogen atoms construct a stable interfacial network that enhances stress transfer and interfacial compatibility [16,30]. This synergy preserves the eco-friendly advantages of waterborne epoxy resin, such as low VOC emissions, while significantly elevating its overall performance through the combined barrier effects of HE-LDHs and the chemical stability of g-C_3_N_4_, yielding exceptional and long-lasting corrosion protection [28,30].

Based on the above analysis, in this study, the magnetic-ultrasonic synergistic dispersion technique, which offers superior dispersion uniformity compared to conventional methods, was employed to physically hybridize HE-LDHs with two-dimensional g-C_3_N_4_, followed by incorporation into a waterborne epoxy resin system. This resulted in the formation of a composite coating with a dual two-dimensional material system (PCN-LDH) for synergistic corrosion protection of Q235 steel. Electrochemical tests revealed that when the mass ratio of platelet g-C_3_N_4_ to HE-LDH was 1:1, the PCN-LDH-1.0 coating exhibited exceptional corrosion resistance. After 28 days of immersion in 3.5% NaCl solution, its coating resistance remained as high as 5.48 × 10^5^ Ω·m^2^, which was five orders of magnitude higher than that of the pristine waterborne epoxy coating, and the corrosion current density was reduced by four orders of magnitude. After a 30-day salt spray test, no rusting or blistering was observed on the surface of the PCN-LDH-1.0 coating, demonstrating excellent durability. Notably, the incorporation of the composite filler did not compromise the mechanical properties of the coating.

## 2. Experimental

### 2.1. Materials

Water epoxy resin (F0704) was purchased from Shenzhen Jitian Chemical Co., Ltd., Shenzhen, China. The resin had an epoxy equivalent weight of 400–800 g/eq, a solid content of 50 ± 3 wt.%, and a rotational viscosity of <1000 mPa·s at 25 °C. The waterborne epoxy curing agent (F0705) was supplied by Shenzhen Jitian Chemical Co., Ltd. Melamine, cyanuric acid, iron(III) nitrate nonahydrate, aluminum nitrate nonahydrate, cobalt(II) nitrate hexahydrate, nickel(II) chloride hexahydrate, zinc chloride, sodium carbonate, sodium hydroxide, anhydrous ethanol, and sodium chloride were supplied by Shanghai Macklin Biochemical Technology Co., Ltd., Shanghai, China. Q235 steel samples (1 cm × 1 cm × 0.3 cm and 12 cm × 5 cm × 0.25 cm) were obtained from Xinhua Metal Products Co., Ltd., Xinyu, China. The chemical composition (wt.%) of the steel was as follows: C ≤ 0.18, Si ≤ 0.25, Mn ≤ 1.20, P ≤ 0.038, S ≤ 0.032, and the balance Fe. The samples were sequentially ground with 200, 800, and 1500-grit sandpapers, followed by ultrasonic cleaning in ethanol and subsequent drying. All chemicals used in the experiments were of analytical grade and were used without further purification.

### 2.2. Preparation of g-C_3_N_4_ Nanosheets and High Entropy Hydroxide

The precursor of g-C_3_N_4_ was synthesized following the reported method [31]. 8 g of melamine and 8 g of cyanuric acid were individually dissolved in 200 mL of dimethyl sulfoxide (DMSO) and stirred in a water bath at 80 °C until complete dissolution, yielding homogeneous transparent solutions. Subsequently, the cyanuric acid solution was slowly added to the melamine solution under continuous stirring. The resulting white precipitate was collected by filtration, washed thoroughly, and dried at 60 °C for 24 h to obtain the g-C_3_N_4_ precursor, marked as PMCS. Then, 2 g of PMCS precursor was placed in a muffle furnace (Shanghai Yiheng Technology Instrument Co., Ltd., Shanghai, China) and heated from room temperature to 520 °C at a rate of 5 °C/min, followed by maintaining the temperature for 2 h to remove impurities and promote crystal structure formation, yielding spherical-aggregated g-C_3_N_4_, marked as QPCN. Finally, 1 g of bulk QPCN was subjected to a second thermal treatment under the same conditions, resulting in well-dispersed g-C_3_N_4_ nanosheets, marked as PCN.

The synthesis of HE-LDHs was prepared by the hydrothermal method as follows. First, Fe(NO_3_)_3_·9H_2_O (2.424 g), Al(NO_3_)_3_·9H_2_O (2.251 g), Co(NO_3_)_2_·6H_2_O (2.328 g), NiCl_2_·6H_2_O (1.902 g) and ZnCl_2_ (1.090 g) were dissolved in 40 mL of deionized water under magnetic stirring to form a metal salt solution. Concurrently, a precipitant solution was prepared by dissolving Na_2_CO_3_ (3.392 g) and NaOH (3.072 g) in 40 mL of deionized water. The two solutions were then added simultaneously into a beaker and mixed with stirring at 1000 rpm, yielding a homogeneous suspension. This suspension was subsequently transferred into a 100 mL Teflon-lined stainless steel autoclave (Xi’an Yichuang Laboratory Instrument Equipment Co., Ltd., Xi’an, China) and subjected to hydrothermal reaction at 80 °C for 48 h in a constant-temperature drying oven (Shaoxing Siyang Instrument Manufacturing Co., Ltd., Shaoxing, China). Finally, the product was washed three times with deionized water and dried at 60 °C for 12 h.

### 2.3. Preparation of Coatings

The HE-LDHs and PCN powders were first dispersed in 2 g of deionized water and subjected to ultrasonic stirring for 20 min. Dispersions with varying amounts of HE-LDHs (0.5 wt.%, 1.0 wt.%, and 1.5 wt.%) and a fixed content of PCN (1 wt.%) were slowly added to the waterborne epoxy resin (WEP) mixture and stirred for 30 min at room temperature. Curing agent was added and stirred uniformly to remove internal bubbles. The resultant coating mixture was evenly sprayed onto the surface of Q235 steel using a spray gun, yielding a coating with an average thickness of 70 ± 5 μm. The composite anticorrosive coatings were obtained by curing at room temperature for 1 day. As a control, pure aqueous epoxy coatings (KB) were prepared following the same procedure. The detailed raw material ratios and coating system nomenclature are presented in Table 1.

### 2.4. Characterization and Testing

The crystal structures of synthesized g-C_3_N_4_ and HE-LDHs nanosheets were characterized by X-ray diffractometer (XRD, Rigaku, Tokyo, Japan) with a scanning range of 10–80° and a scanning rate of 8°·min^−1^. The functional groups of the prepared nanomaterials and composite coatings were analyzed using Fourier transform infrared spectrometer (FT-IR, ALPHA, Bruker, Billerica, MA, USA) in the spectral range of 400–4000 cm^−1^. The morphologies of HE-LDHs and the cross-section of composite coatings were observed by scanning electron microscopy (SEM, SU8010, Hitachi, Tokyo, Japan). The microstructure of HE-LDHs was investigated by transmission electron microscopy (TEM, JEM2100F, JEOL, Tokyo, Japan) to obtain the morphologies of the top surface and cross-section of the composite coating. The corrosion resistance of the composite coating was further tested by neutral salt spray test (KeHeng Testing Instruments, Shenzhen, China) following ASTM B117 standard. The thickness of the composite coating was measured using a coating thickness gauge (Guangzhou Guoou Electronic Technology, Guangzhou, China). A tool was used to scratch the coating surface, and then 3M tape was attached to the cut surface and removed after 90 s.

The corrosion resistance and immersion performance of pure epoxy and composite coatings in 3.5 wt.% NaCl solution were evaluated using CHI760E electrochemical workstation (Shanghai Chenhua Instruments, Shanghai, China). Electrochemical impedance spectroscopy (EIS) and potentiodynamic polarization measurements were conducted in a three-electrode system, where the sample served as the working electrode, platinum electrode as the counter electrode, and saturated calomel electrode (SCE) as the reference electrode. The tests were performed in 3.5 wt.% NaCl solution, with multiple time intervals for assessing the corrosion resistance stability of the coatings. The alternating current (AC) amplitude was set at 10 mV, and the frequency range was from 1 × 10^−2^ to 1 × 10^5^ Hz. The polarization curves were scanned from 200 mV below the open circuit potential (OCP) to 200 mV above the OCP at a scan rate of 1 mV·s^−1^. Additionally, three representative samples were immersed in the corrosive solution for 28 days, and Tafel tests were conducted after the potential stabilized to investigate the corrosion potential (E_corr_) and corrosion current density (i_corr_), which were calculated by Tafel extrapolation method [32]. Corrosion rate (V_corr_, in millimeters per year) was calculated from the following Equation (1):

(1) $$V_{corr\;}\left(mm\;{year}^{-1}\right)=\frac{i_{corr\;}(A/m^{2})\;M\;(g)}{D\;(g{/cm}^{3})\;V}\times 0.327$$

where i is the current (A/m^2^), M is the molecular weight, V is valence, 0.327 is constant and D is the density (g/cm^3^).

The corrosion resistance of the composite coating was further tested by neutral salt spray test (KeHeng Testing Instruments, China) following ASTM B117 standard [33].

## 3. Results and Discussion

### 3.1. Characterization of HE-LDHs

**Figure 1:**
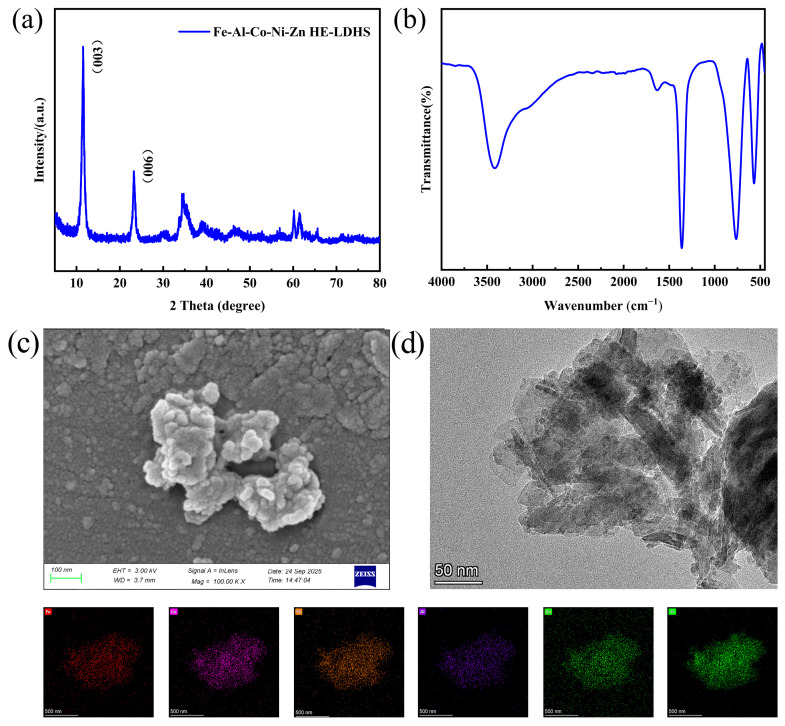
Structural characterization of HE-LDHs samples: (**a**) XRD patterns; (**b**) FT-IR spectra; (**c**) SEM image; (**d**) TEM image and EDS mapping.

Figure 1a presents the XRD pattern of the synthesized HE-LDHs. The characteristic diffraction peaks observed at 11.7° and 23.5° correspond to the (003) and (006) crystal planes of the LDH structure, respectively, confirming the successful formation of a characteristic layered structure [34]. The presence of the (012) plan diffraction peak at 34.7° further substantiates the formation of the LDH phase [35]. The sharp and symmetric nature of these diffraction peaks indicates that the as-synthesized HE-LDHs possess high crystallinity and adopt the typical crystal structure of conventional LDH materials [36]. The interlayer spacing (d003) calculated using Bragg equation from the (003) diffraction peak is approximately 0.76 nm. This value aligns well with reported data for LDHs intercalated with carbonate anions [37], validating the structural regularity and successful carbonate intercalation within the HE-LDHs galleries. Figure 1b shows the FT-IR spectrum of HE-LDHs. The broad and intense absorption band centered around 3440 cm^−1^ is attributed to the O-H stretching vibration of hydroxyl groups in the brucite-like layers and interlayer water molecules, confirming the formation of the characteristic layered hydroxide structure [38]. The sharp peak near 1365 cm^−1^ corresponds to the v3 asymmetric stretching vibration of carbonate ions (CO_3_^2−^), indicating that CO_3_^2−^ is the predominant intercalated anion. This is consistent with the known tendency of LDHs to incorporate carbonate from atmospheric CO_2_ during synthesis or handling, which often contributes to their structural stability [39]. The vibrational bands in the region of 500–600 cm^−1^ are assigned to the lattice vibrations of M–O–M (e.g., Fe–O, Co–O, Ni–O), within the octahedral sheets [40]. Additionally, the absorption feature near 750 cm^−1^ may be associated with bending vibrations of Al–O and Zn–O bonds, supporting the incorporation of these metals into the layered structure [41].

The morphology of the synthesized HE-LDHs was examined by SEM. As shown in Figure 1c, the sample exhibits a typical two-dimensional layered nanostructure, which closely resembles the morphological features of conventional LDHs. HE-LDHs nanosheets are stacked into a flower-like hierarchical architecture without obvious aggregation, indicating the formation of a layered morphology characteristic of LDHs materials. This hierarchical flower-like architecture, composed of ultrathin nanosheets, is anticipated to facilitate a uniform dispersion within the polymer matrix and create a highly tortuous diffusion path for corrosive species, which is crucial for enhancing the barrier property of the composite coating [42]. TEM was further employed to investigate the microstructure. As presented in Figure 1d, the image shows transparent, single-layer or few-layer sheet nanostructure, further confirming the typical layered nanosheet morphology of HE-LDHs and agreeing well with the SEM observations. EDS mapping confirmed the homogeneous distribution of all metallic constituents (Fe, Co, Ni, Al, Zn) and oxygen at the micron scale, indicating the successful incorporation of multiple elements into the LDH structure without macroscopic phase segregation. These EDS findings are consistent with the XRD results (single LDH phase) and FT-IR data (characteristic metal-oxygen vibrations), collectively supporting the successful synthesis of a multi-metal incorporated LDH.

**Figure 2:**
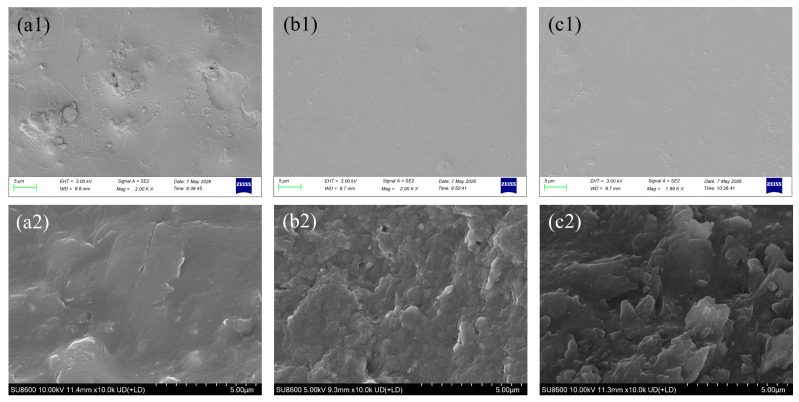
Surface and cross-sectional SEM images of blank coating (**a1**,**a2**); PCN-1.0 coating (**b1**,**b2**) and PCN-LDH-1.0 coating (**c1**,**c2**).

The surfaces and cross-sectional morphologies of KB, PCN-1.0 and PCN-LDH-1.0 coatings were examined by SEM to assess their microstructural integrity and filler dispersion, as shown in Figure 2. Cross-sectional samples were prepared by cryo-fracturing in liquid nitrogen. The surface of the KB coating (Figure 2a1) displayed numerous micropits and irregularities, likely arising from solvent evaporation and/or volumetric shrinkage during epoxy curing [15]. Its cross-section (Figure 2a2) showed a relatively smooth fracture surface with minimal plastic deformation, characteristic of brittle fracture in thermosetting polymers [43]. Moreover, micron-sized voids and microcracks were present, which could serve as channels for corrosive electrolyte penetration, detrimentally affecting the barrier property of coatings. In contrast, the PCN-1.0 coating surface (Figure 2b1) showed smoother but contained some aggregated particles, presumably g-C_3_N_4_ nanosheets. The corresponding cross-section (Figure 2b2) exhibited a rougher fracture surface with pronounced river patterns and shear ridges, suggesting increased energy dissipation during fracture and a transition towards more ductile behavior compared to the KB coating [17]. This indicates that g-C_3_N_4_ incorporation enhanced the toughness of the epoxy matrix. However, a limited number of microporous defects were also observed, likely due to imperfect interfacial adhesion between g-C_3_N_4_ and the epoxy, leading to filler aggregation and void formation under stress [44].

Remarkably, the PCN-LDH-1.0 coating exhibited the most uniform morphology. Its surface (Figure 2c1) was very smooth and homogeneous, with no visible pits or protruding aggregates. The cross-section (Figure 2c2) displayed abundant shear wrinkles and was devoid of the microporous defects seen in PCN-1.0, indicating a denser, more coherent microstructure with excellent filler-matrix integration. This superior morphology is attributed to the synergistic effect between HE-LDHs and g-C_3_N_4_. The lamellar HE-LDHs likely act as a dispersion aid for g-C_3_N_4_ nanosheets, while their interfacial interactions (e.g., hydrogen bonding) enhance compatibility with the epoxy matrix [17]. Consequently, the uniformly dispersed hybrid fillers and the densified interface are expected to create an effective “tortuous path” barrier, significantly impeding the penetration of corrosive species and contributing to the enhanced corrosion resistance demonstrated by this composite coating.

### 3.2. Corrosion Behavior of Coatings in 3.5 wt.% NaCl Solution

**Figure 3:**
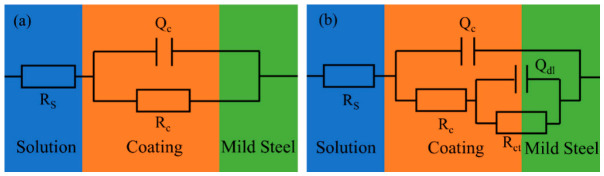
Equivalent circuit models for fitting raw EIS data: (**a**) one time constant; (**b**) two time constants.

**Figure 4:**
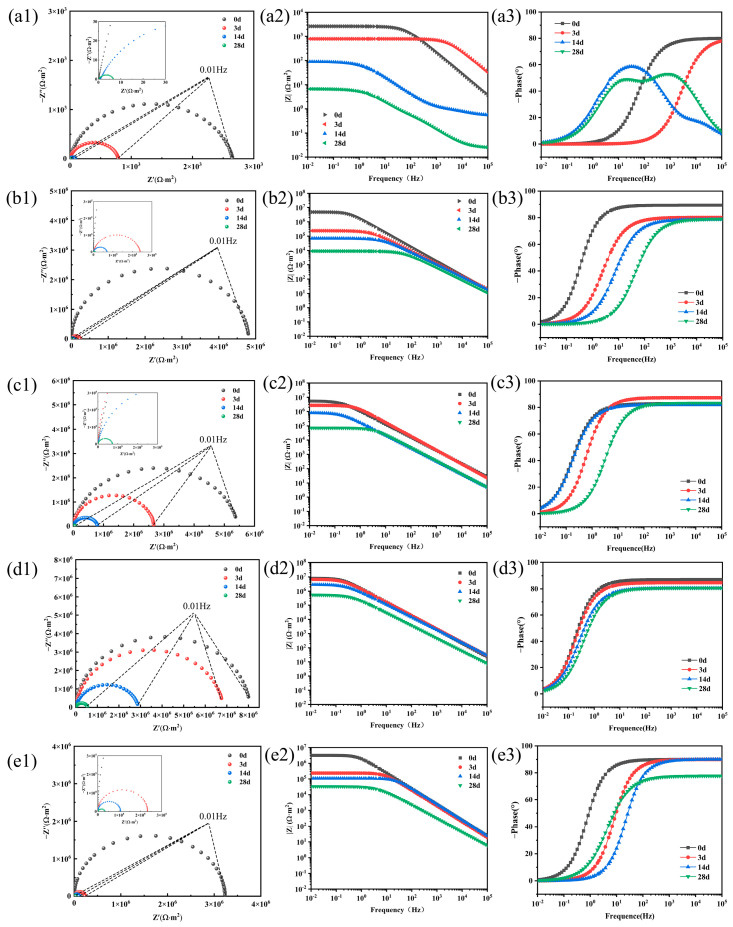
Nyquist, Bode, and phase angle plots for different coatings after immersion in 3.5 wt.% NaCl solution for a different time. (**a1**–**a3**) WEP coating; (**b1**–**b3**) PCN-1 coating; (**c1**–**c3**) PCN-LDH-0.5 coating; (**d1**–**d3**) PCN-LDH-1.0 coating; (**e1**–**e3**) PCN-LDH-1.5 coating.

EIS was employed to evaluate the corrosion resistance of the different coatings, as it is a sensitive technique for monitoring the degradation and failure processes of coatings in laboratory settings [43,45,46]. During the test, all coatings were immersed in 3.5 wt.% NaCl solution, with immersion intervals set at 0 (initial), 3, 14, and 28 days. To quantitatively analyze the EIS data, appropriate equivalent circuit models were established, as shown in Figure 3. In the initial immersion stage, where the coating acts as an effective barrier, the impedance behavior can be modeled using a simple R(QR) circuit [47] (as shown in Figure 3a). This circuit comprises the solution resistance (R_s_), coating resistance (R_c_), and a constant phase element (CPE_c_) representing the non-ideal capacitive behavior of the protective coating [44]. As the test proceeds, when the corrosive medium penetrates through the protective layer of the coating and reaches the metallic substrate, electrochemical corrosion is triggered [48]. The CPE is used to account for surface heterogeneity and frequency dispersion effects. As immersion time increases, the penetration of electrolyte through coating defects may reach the metal substrate, initiating electrochemical corrosion reactions at the interface. This process introduces a second time constant in the impedance spectrum. To model this scenario, a more complex R(QR) circuit (Equivalent Circuit B, Figure 3b) was employed. This circuit introduces additional parameters: the charge transfer resistance (R_ct_) associated with the corrosion reaction, and the corresponding double-layer capacitance (CPE_dl_) at the metal/solution interface [49]. As shown in Figure 4, the measured EIS data were fitted using ZView 4.1b software and plotted as Nyquist and Bode diagrams [50]. Table 2 provides a detailed account of the fitted parameters of EIS data for KB, PCN-1, PCN-LDH-0.5, PCN-LDH-1.0, and PCN-LDH-1.5 coatings immersed in 3.5 wt.% NaCl solution.

Figure 4a1–e1 shows the Nyquist plots of different coatings after immersion in 3.5 wt.% NaCl solution for periods up to 28 days. At the initial immersion stage (0 days, 1 h), the Nyquist plot of the KB coating exhibited a single, depressed capacitive arc. This is characteristic of a barrier-type coating, where the electrochemical response is dominated by the capacitance and pore resistance of the intact coating, indicating that the electrolyte had not yet penetrated to the metal substrate [43]. This suggests that the coating acted as an effective barrier at this stage, with minimal penetration of corrosive species to the Q235 steel substrate, thus preventing detectable electrochemical corrosion. According to the literature [51,52], the diameter of the capacitive arc in the high-frequency region is often correlated with the pore resistance of the coating, which is a direct measure of its barrier property against electrolyte ingress. A larger arc diameter signifies higher R_c_ and thus better corrosion protection performance. As expected, the diameter of the capacitive arcs for all coatings decreased with prolonged immersion time, indicating a progressive degradation of their barrier properties. This is attributed to the uptake of water and chloride ions into coating matrix, which can plasticize the polymer, swell the network, and eventually form conductive pathways, thereby reducing the coating’s electrical resistance and barrier integrity [51]. After 14 days of immersion, the Nyquist plot of the KB coating evolved to display two distinct time constants (two depressed semicircles). This evolution signifies that the electrolyte has penetrated through the coating defects and reached the Q235 steel substrate, initiating electrochemical corrosion reactions at the coating/metal interface. The high-frequency arc is associated with the coating properties, while the newly emerged low-frequency arc corresponds to the charge R_ct_ of the corrosion process [48]. In stark contrast, all nanocomposite coatings (PCN-1.0 and PCN-LDH series) maintained a single capacitive arc at this stage. More importantly, the diameter of these arcs remained significantly larger than that of the KB coating even at 0 days, quantitatively demonstrating their superior barrier property. Notably, the PCN-LDH-1.0 coating exhibited the largest arc diameter among all modified coatings, suggesting the most effective resistance to electrolyte penetration. After 28 days, the Nyquist plot of the KB coating showed a second time constant, confirming that the electrolyte had penetrated to the substrate and sustained corrosion was occurring. Conversely, all nanofiller-modified coatings retained a single, albeit smaller, capacitive arc throughout the 28-day test, indicating that the coating barrier property remained the dominant factor controlling the impedance response. Most notably, the PCN-LDH-1.0 coating consistently exhibited the largest capacitive arc diameter at all immersion times, implying the best long-term barrier performance. The synergistic effect between g-C_3_N_4_ expansion diffusion pathway and HE-LDHs inhibition of electrochemical activity effectively delays the penetration of corrosive media [2].

Figure 4a2–e2 and Figure 4a3–e3 show the Bode magnitude and phase angle plots, respectively. The low-frequency impedance modulus (|Z|0.01 Hz), which is indicative of the overall corrosion resistance, decreased for all coatings with immersion time. Concurrently, the maximum phase angle decreased, and its peak frequency increased, suggesting a deterioration of the coatings’ capacitive barrier behavior and the possible initiation of charge-transfer processes at the interface. Specifically, the |Z|0.01 Hz of the KB coating decreased from 2.65 × 10^3^ Ω·m^2^ to 6.70 × 10^0^ Ω·m^2^, and that of the PCN-1.0 coating dropped from 4.80 × 10^6^ Ω·m^2^ to 9.08 × 10^3^ Ω·m^2^. Notably, as the HE-LDH dosage increases, the |Z|0.01 Hz of the coatings first increases and then decreases. After 28 days of immersion, the |Z|0.01 Hz of the PCN-LDH-1.0 coating remained at 5.43 × 10^5^ Ω·m^2^, which is 200 times higher than the initial |Z|0.01 Hz of the KB coating. The initial |Z|0.01 Hz of PCN-LDH-1.0 is over 3 orders of magnitude higher than that of the pure KB coating. This remarkable enhancement can be directly correlated with the dense and defect-free microstructure observed in SEM (Figure 2c2), where the HE-LDHs and g-C_3_N_4_ construct an intricate labyrinthine structure that drastically extends the diffusion pathway for corrosive agents. More importantly, it shows a much slower degradation rate compared to other coatings. Combined with its highest absolute impedance value among all samples after long-term exposure, conclusively demonstrates the superior long-term corrosion protection efficacy of the PCN-LDH-1.0 composite coating.

As shown in Table 2, prolonged immersion in the 3.5 wt.% NaCl solution resulted in a drastic degradation of the electrochemical parameters for the pure KB coating. Specifically, the coating resistance (R_c_) of the KB coating decreased sharply from 2.65 × 10^3^ Ω·m^2^ to 9.78 × 10^−1^ Ω·m^2^ after 28 days of immersion. Concurrently, the constant phase element parameter (CPE_c_, represented Y_0_) for the coating increased significantly from 1.91 × 10^−5^ Ω^−1^·m^−2^·s^n^ to 9.97 × 10^−3^ Ω^−1^·m^−2^·s^n^. The increase in Y_0_ value is typically associated with water uptake and the formation of conductive pathways within the coating matrix [48]. Generally, a decrease in R_c_ coupled with an increase in CPE_c_ (Y_0_) signifies the deterioration of the coating’s barrier property. This is due to the uptake of electrolyte, which plasticizes the polymer, increases the dielectric constant, and eventually establishes conductive paths, facilitating the transport of corrosive species towards the metal substrate [4]. When the EIS spectrum exhibits two time constants, a decrease in the R_ct_ and an increase in the double layer CPE parameter (CPE_dl_, Y_0_) indicate an acceleration of the electrochemical corrosion reaction at the coating/metal interface [43]. The KB coating shows a double-time-constant characteristic after only 14 days of immersion, suggesting a sharp decline in its corrosion resistance. In contrast, both the PCN-1.0 and the PCN-LDH-X series coatings maintained R_c_ values that were orders of magnitude higher and CPE_c_ values that were orders of magnitude lower than those of the KB coating at each immersion interval. This indicates a vastly superior and more stable barrier property. This phenomenon is attributed to the introduction of g-C_3_N_4_ and HE-LDHs nanofillers. These fillers enhance the barrier efficiency by creating a more tortuous path, which significantly hinders the penetration of water and aggressive ions through the coating matrix [2]. Further comparative analysis shows that within the same immersion period, all PCN-LDH series coatings have higher R_c_ values and lower CPE_c_ values than the PCN-1.0 coating. The corrosion resistance of the PCN-LDH coatings shows a non-monotonic trend with increasing HE-LDH content, peaking at 1.0 wt.%. This optimal performance is attributed to a synergistic effect between HE-LDHs and g-C_3_N_4_. The layered structure of HE-LDHs may act as a scaffold to improve the dispersion of g-C_3_N_4_ nanosheets, while both components together enhance the interfacial adhesion with the epoxy matrix, constructing a more effective barrier network. However, beyond the optimal loading (e.g., 1.5 wt.%), nanoparticle aggregation likely occurs, introducing defects that compromise the coating integrity and reduce the barrier performance [25]. Even after 28 days of immersion, the PCN-LDH-1.0 coating remains an R_c_ value as high as 5.48 × 10^5^ Ω·m^2^. Its Rc retention rate (R_c_, 28 d/R_c_, 0 d) was significantly higher than that of the PCN-1.0 and KB coatings, demonstrating exception long-term stability. These results demonstrate that the optimal combination of g-C_3_N_4_ and HE-LDHs (1.0 wt.%) creates a highly tortuous path within the epoxy matrix, which dramatically delays the ingress of corrosive media [53]. Consequently, the PCN-LDH-1.0 coating exhibits significantly superior and more durable corrosion resistance compared to both the pure KB coating and the singly modified PCN-1.0 coating, confirming an ideal long-term barrier effect.

**Figure 5:**
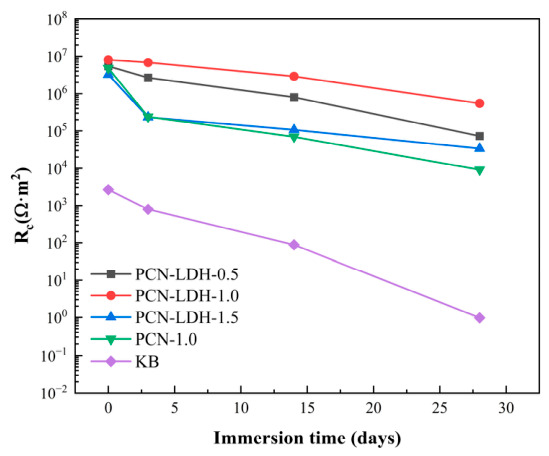
Evolution of coating resistance Rc of different coatings during immersion in 3.5 wt.% NaCl solution at room temperature.

Figure 5 illustrates the evolution of coating R_c_ for all samples over the 28-day immersion period in 3.5 wt.% NaCl solution at room temperature. The PCN-LDH-1.0 coating exhibited the highest initial R_c_ value, approximately 3 orders of magnitude greater than that of the KB coating, and maintained this superior performance advantage throughout the entire test. This outstanding and persistent barrier property is attributed to the optimal dispersion of the g-C_3_N_4_/HE-LDHs hybrid filler, which creates a highly tortuous path and strong interfacial bonding within the epoxy matrix, as evidenced by the dense microstructure observed in SEM (Figure 2). As expected, the R_c_ values for all coatings decreased progressively with immersion time. This universal decline signifies the gradual deterioration of the coatings’ barrier properties, primarily due to the uptake of water and chloride ions. Most notably, even after the 28-day immersion, the PCN-LDH-1.0 coating not only retained the highest absolute R_c_ value but also exhibited the smallest relative decrease, indicating exceptional long-term stability. This optimal performance at 1.0 wt.% HE-LDHs loading highlights the existence of a synergistic threshold, beyond which (as seen with PCN-LDH-1.5) nanoparticle aggregation likely compromises the barrier network. Therefore, the R_c_ evolution data conclusively demonstrate that the PCN-LDH-1.0 formulation provides the most effective and durable corrosion protection among all tested coatings.

**Figure 6:**
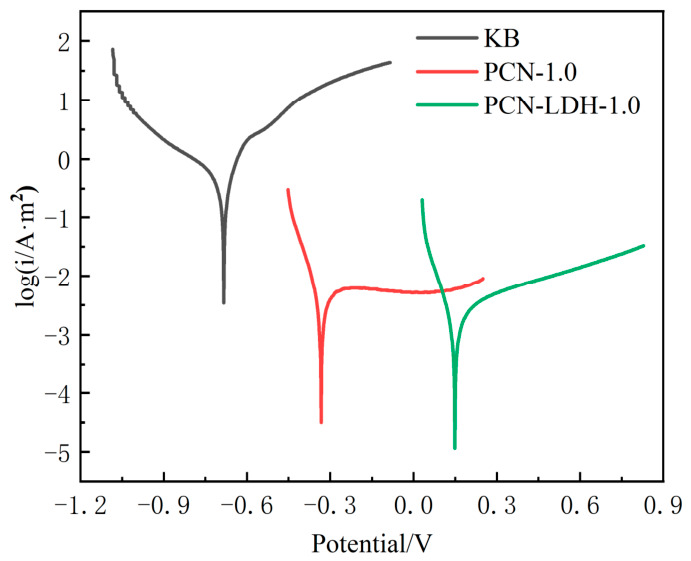
Polarization curves of coatings immersed in 3.5 wt.% NaCl solution for 28 days.

The long-term anti-corrosion performance was further assessed via potentiodynamic polarization tests after 28 days of immersion in 3.5 wt.% NaCl solution (simulating a marine environment). Figure 6 presents the polarization curves for three representative coatings: the KB, PCN-1.0 and PCN-LDH-1.0 coatings. The corresponding corrosion potential (E_corr_) and corrosion current density (i_corr_) derived from Tafel extrapolation are listed in Table 3. In coated systems, a more positive Ecorr may suggest a shift in the corrosion thermodynamics, but it should be interpreted with caution, as it can be influenced by mixed electrode processes under the coating. A more direct and reliable indicator of protection is the i_corr_, where a lower value signifies a slower electrochemical corrosion rate [9]. The KB coating exhibited the most negative E_corr_ (−0.684 V) and the highest i_corr_ (5.83 × 10^−1^ A·m^−2^), consistent with its poor barrier property revealed by EIS, indicating severe corrosion activity on the exposed substrate. Incorporation of g-C_3_N_4_ (PCN-1.0 coating) reduced the i_corr_ by over two orders of magnitude to 3.47 × 10^−3^ A·m^−2^. This dramatic reduction is attributed to the barrier effect of the two-dimensional g-C_3_N_4_ nanosheets, which create a tortuous path that significantly hinders the penetration of corrosive species to the steel substrate [32]. Notably, the PCN-LDH-1.0 coating demonstrated a further remarkable result that its i_corr_ decreased to an exceptionally low value of 1.68 × 10^−5^ A·m^−2^, and its E_corr_ shifted positively to 0.148 V. The calculated corrosion rate (V_corr_) for PCN-LDH-1.0 coating was merely 1.957 × 10^−5^ mm·year^−1^. This value is four orders of magnitude lower than that of the KB coating and two orders of magnitude lower than that of the PCN-1.0 coating, providing a compelling quantitative comparison of the performance enhancement. These polarization results, consistent with the EIS data, unequivocally show that the g-C_3_N_4_/HE-LDHs filler endows the epoxy coating with superior corrosion protection. The drastically reduced i_corr_ and V_corr_ of the PCN-LDH-1.0 coating suggest that the combination of g-C_3_N_4_ and HE-LDHs creates a synergistic effect, resulting in an exceptionally efficient and durable protective barrier for the Q235 steel substrate. Compared with other modified anti-corrosion coatings, PCN-LDH-1.0 shows excellent anti-corrosion performance (Table 4).

### 3.3. Coating Mechanical Properties

The mechanical properties of coatings, such as hardness, adhesion strength, and impact resistance, are critical parameters for evaluating their protective performance and practical application potential [43]. Among these, adhesion strength, which directly reflects the coating-substrate interfacial bonding quality, is of particular importance for assessing coating stability under complex service conditions, as it significantly influences the coating’s service life and protective efficiency [58].

Table 5 reveals significant differences in the mechanical properties among the coatings. Notably, the PCN-1.0, PCN-LDH-0.5, and PCN-LDH-1.0 coatings demonstrate the best overall mechanical performance. These three coatings show comparable and excellent performance in hardness, adhesion strength, and impact resistance, with their values being significantly higher than those of the KB coating. This enhancement in mechanical properties can be attributed to the formation of a denser and more uniform microstructure, as evidenced by the SEM analysis (Figure 2). This improved microstructure effectively enhances the resistance to plastic deformation, promotes stronger interfacial adhesion between the coating and the substrate, and improves energy dissipation capability under sudden load [3]. The fact that the singly modified PCN-1.0 coating exhibits superior properties suggests that g-C_3_N_4_ itself acts as an effective reinforcing filler. Furthermore, the optimal performance within the PCN-LDH series at 0.5–1.0 wt.% HE-LDHs loading implies a potential synergistic effect between g-C_3_N_4_ and HE-LDHs in optimizing the composite microstructure and stress transfer, leading to the best overall mechanical performance.

### 3.4. Salt Spray Test

**Figure 7:**
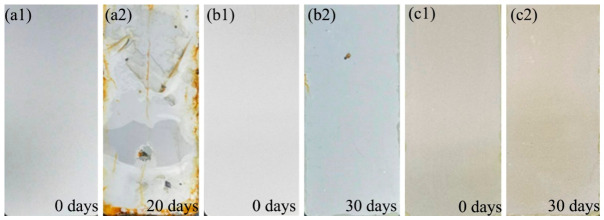
Digital images of the salt spray test coatings. (**a1**,**a2**) KB coating; (**b1**,**b2**) PCN-1.0 coating; (**c1**,**c2**) PCN-LDH-1.0 coating.

**Figure 8:**
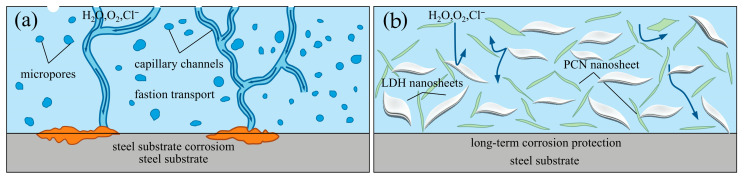
Schematic diagram of anti-corrosion mechanism of composite coating. (**a**) KB coating; (**b**) PCN-LDH coating.

The salt spray test is a critical accelerated method for evaluating the long-term corrosion resistance of coatings, as it effectively simulates harsh marine or high-salinity industrial atmospheric environments [59]. In this work, to evaluate the protective performance of various coatings on Q235 steel, coated specimens were subjected to a continuous 5 wt.% NaCl salt spray test for 30 days. The macroscopic morphologies of the coatings before and after the salt spray test are compared in Figure 7. The pure epoxy (KB) coating (Figure 7a2) showed early failure, exhibiting severe blistering and delamination from the substrate after only 20 days of exposure, accompanied by extensive red rust formation. This indicates that the barrier function of the KB coating was completely compromised, allowing corrosive media to penetrate and initiate electrochemical corrosion on the steel substrate, as schematically illustrated in Figure 8a. In contrast, the coating modified with g-C_3_N_4_ (PCN-1.0, Figure 7b2) demonstrated markedly improved resistance, showing only minor surface blistering and no visible corrosion products after the full 30-day test. The composite coating incorporating both g-C_3_N_4_ and HE-LDHs (exemplified by the optimal PCN-LDH-1.0 formulation from electrochemical tests) exhibited the best performance. Even after the full 30-day test, it remained largely intact (Figure 7c2), with no visible blistering, cracking, or rust spots, corresponding to the highest protection rating.

### 3.5. Anti-Corrosion Mechanism

The exceptional corrosion resistance of the PCN-LDH composite suggests a synergistic effect between the two nanofillers. This improvement can be attributed to the well-dispersed two-dimensional g-C_3_N_4_ nanosheets, which create a tortuous path effect that significantly hinders and prolongs the diffusion of corrosive species (H_2_O, O_2_, Cl^−^) through the coating matrix, thereby enhancing its barrier efficiency [60]. This synergy likely stems from their complementary barrier functions: the g-C_3_N_4_ nanosheets provide a primary tortuous path, while the HE-LDHs further refine the pore structure and may plug defects, collectively creating a more efficient and hierarchical barrier network against corrosive species [61], as illustrated in Figure 8b. Beyond the physical barrier effect, HE-LDHs may also contribute to active corrosion protection [62]. Similarly to conventional LDHs, the anions intercalated within the HE-LDHs galleries could be released upon contact with corrosive electrolytes. These released anions might then migrate to the coating-metal interface, potentially inhibiting the anodic dissolution of the steel substrate [63].

## 4. Conclusions

In summary, we successfully prepared a high-performance waterborne epoxy coating by incorporating a hybrid filler composed of HE-LDHs and g-C_3_N_4_ for the corrosion protection of Q235 steel. EIS and potentiodynamic polarization measurements revealed that the PCN-LDH-1.0 coating with a g-C_3_N_4_ to HE-LDHs mass ratio of 1:1 demonstrated the best corrosion protection performance among all coatings. After 28 days of immersion in 3.5 wt.% NaCl solution, the PCN-LDH-1.0 coating maintained a high R_c_ of 5.48 × 10^5^ Ω·m^2^, along with a low corrosion current density of 1.68 × 10^−5^ A·m^−2^ and corrosion rate of 1.957 × 10^−5^ mm·year^−1^. The long-term protective capability was further corroborated by a 30-day neutral salt spray test, in which the PCN-LDH-1.0 coating showed no signs of blistering, rust, or adhesion loss. The g-C_3_N_4_ nanosheets and HE-LDHs primarily enhance the physical barrier property by creating a tortuous path for corrosive species. Moreover, the PCN-LDH-1.0 coating retained excellent mechanical properties, including a pencil hardness of 3H and the highest adhesion grade, which are crucial for maintaining coating integrity under mechanical stress and ensuring its practical application potential. This study provides a simple and cost-effective approach for developing high-performance waterborne epoxy anti-corrosion coatings.

## Figures and Tables

**Table 1:** Raw material ratios for different coatings.

Coatings	WEP (g)	Curing Agent (g)	Deionized Water (g)	PCN (g)	HE-LDHs (g)
**KB**	10	5	2	0	0
**PCN-1.0**	10	5	2	0.1	0
**PCN-LDH-0.5**	10	5	2	0.1	0.05
**PCN-LDH-1.0**	10	5	2	0.1	0.1
**PCN-LDH-1.5**	10	5	2	0.1	0.15

**Table 2:** Fitted electrochemical parameters based on equivalent circuit modeling of EIS data.

Coatings		CPE_c_		Rc (Ω·m^2^)	CPE_dl_		R_ct_ (Ω·m^2^)
		Y_0_ (Ω^−1^·m^−2^·s^n^)	n_coat_		Y_0_ (Ω^−1^·m^−2^·s^n^)	n_dl_	
**KB**	0 d	1.91 × 10^−5^	0.8986	2.65 × 10^3^			
	3 d	5.06 × 10^−4^	0.8924	7.89 × 10^2^			
	14 d	4.89 × 10^−5^	0.7525	1.15 × 10^0^	1.47 × 10^−3^	0.7668	8.84 × 10^1^
	28 d	9.97 × 10^−3^	0.7493	9.78 × 10^−1^	1.28 × 10^−2^	0.7822	5.75 × 10^0^
**PCN-1.0**	0 d	9.59 × 10^−7^	0.9929	4.80 × 10^6^			
	3 d	3.58 × 10^−7^	0.8903	2.40 × 10^5^			
	14 d	4.97 × 10^−6^	0.8735	6.96 × 10^4^			
	28 d	7.65 × 10^−5^	0.8782	9.08 × 10^3^			
**PCN-LDH-0.5**	0 d	1.70 × 10^−7^	0.9801	5.45 × 10^6^			
	3 d	1.03 × 10^−7^	0.9716	2.67 × 10^6^			
	14 d	1.09 × 10^−6^	0.9403	8.08 × 10^5^			
	28 d	8.37 × 10^−7^	0.9268	7.29 × 10^4^			
**PCN-LDH-1.0**	0 d	1.08 × 10^−7^	0.9673	8.07 × 10^6^			
	3 d	1.22 × 10^−7^	0.9413	6.84 × 10^6^			
	14 d	2.20 × 10^−7^	0.8948	2.91 × 10^6^			
	28 d	8.23 × 10^−7^	0.8964	5.48 × 10^5^			
**PCN-LDH-1.5**	0 d	6.66 × 10^−8^	0.9774	3.23 × 10^6^			
	3 d	8.51 × 10^−8^	0.9681	2.34 × 10^5^			
	14 d	6.65 × 10^−8^	0.9165	1.07 × 10^5^			
	28 d	1.67 × 10^−6^	0.8634	3.37 × 10^4^			

**Table 3:** Fitted electrochemical parameters based on polarization curves.

Coatings	Ecorr (V)	Icorr (A m^−2^)	Vcorr (mm year^−1^)
KB	−0.684	5.83 × 10^−1^	6.778 × 10^−1^
PCN-1.0	−0.333	3.47 × 10^−3^	4.038 × 10^−3^
PCN-LDH-1.0	0.148	1.68 × 10^−5^	1.957 × 10^−5^

**Table 4:** Anticorrosion performance between this work and other works.

Coating	Matrix	Filling	Time	|Z|0.01 Hz(Ω·m^2^)	Ref
KB	water epoxy resin	-	28 d	6.70 × 10^0^	This work
PCN-LDH-1.0	water epoxy resin	PCN-LDH	28 d	5.43 × 10^5^	
GO-3/WEP	water epoxy resin	GO	15 d	5.82 × 10^4^	[54]
0.50 wt.% MXene/PEI/WEP	water epoxy resin	MXene/PEI	30 d	8.29 × 10^4^	[55]
CML/WEP	water epoxy resin	CML	28 d	3.72 × 10^4^	[56]
g-C_3_N_4_@GO/WEC	water epoxy resin	g-C_3_N_4_@GO	40 d	8.37 × 10^3^	[16]
HNT-CS@BTA	Epoxy E44	HNT-CS@BTA	42 d	1.47 × 10^5^	[57]

**Table 5:** Mechanical properties of different coatings.

Coatings	Hardness	Adhesive Force	Impact Resistance (cm)
KB	2H	0	50
PCN-1.0	3H	0	50
PCN-LDH-0.5	3H	0	50
PCN-LDH-1.0	3H	0	50
PCN-LDH-1.5	H	2	40

## Data Availability

The original contributions presented in this study are included in the article. Further inquiries can be directed to the corresponding authors.
